# Intervention through an intelligent technological platform for socio-emotional development and health promotion in adolescents with Type 1 Diabetes Mellitus (emoTICare): A study protocol for randomized controlled trial

**DOI:** 10.1371/journal.pone.0325763

**Published:** 2025-06-23

**Authors:** Javier Martín-Ávila, Esther Rodríguez-Jiménez, Selene Valero-Moreno, José-Antonio Gil-Gómez, Inmaculada Montoya-Castilla, Marián Pérez-Marín

**Affiliations:** 1 Departamento de Personalidad, Evaluación y Tratamientos Psicológicos, Facultad de Psicología y Logopedia, Universitat de València, Valencia, Spain; 2 Instituto Universitario de Investigación en Automática e Informática Industrial, Universitat Politècnica de València, Valencia, Spain; Federal University of Ceara, BRAZIL

## Abstract

**Background:**

During adolescence, individuals face various challenges that require adequate psychosocial and emotional adjustment. Additionally, the presence of a chronic illness during this stage, such as Type 1 Diabetes Mellitus (T1DM), introduces additional medical and psychological needs, which can increase vulnerability. In this context, serious games emerge as a promising tool to address educational and psychoemotional aspects while maintaining the motivation and playability typical of a video game. Currently, no technological tool simultaneously addresses both the educational and psycho-emotional needs of adolescents with T1DM.

**Aim:**

This study aims to design and validate a psychological intervention program using a technological platform that incorporates artificial intelligence. Through the serious game emoTICare, the goal is to promote physical and emotional health in adolescents with T1DM by enhancing the development of socio-emotional skills.

**Method:**

Test this tool, adolescents with T1DM will be recruited through diabetes associations across Spain. The participants, 372 adolescents aged between 12 and 16, will be randomly assigned to either a control or an experimental group. The experimental group will complete the emoTICare program. Over six weeks, participants will engage in psychoeducational activities using the tools provided by the serious game. Data Will be collected at three points: baseline (T1) and after six weeks (T2) (for the control group, T2 will be at the same time as the experimental group’s post-intervention), and aT3 (after six months of T2). The program’s effectiveness will be analyzed using various statistical packages.

**Results and conclusions:**

The study hypothesizes that adolescents will maintain or improve their physical and emotional health indicators following the emoTICare program. Specifically, statistically significant improvements (p ≤ .05) are expected in the following variables: health-related quality of life, social skills and communication, emotional awareness, resilience, self-esteem, and coping and problem-solving abilities. Additionally, a statistically significant reduction (p ≤ .05) is anticipated in variables related to the perceived threat of the disease, as well as emotional and behavioral problems. The experimental group is expected to present improvements in all these areas when comparing pre- and posttreatment evaluations, and also in comparison to the control group on the waiting list.

## Introduction

Adolescence is a developmental stage characterised by significant physiological, social and psychological transformations [1]. This period is characterised by a combination of challenges and opportunities, as young individuals commence the process of defining their identities [2] and assuming an increasing array of responsibilities [3,4]. These transitions can be particularly challenging for adolescents [5,6], with the potential to have a significant impact on their quality of life and psychological well-being [7].

The presence of a chronic illness (CI) can further compound the challenges faced by adolescents [8,9]. According to the World Health Organization (WHO), the most prevalent chronic diseases include cardiovascular conditions, cancer, chronic respiratory diseases, and diabetes [10]. Of these, Type 1 Diabetes Mellitus (T1DM) is a particular focus, given its propensity to emerge during childhood and adolescence, although it can develop at any age [11]. T1DM is the most prevalent chronic endocrine disorder during childhood [12–15] and remains one of the most frequent chronic illnesses in adolescence [16–18]. The incidence of T1DM is highest during preadolescence and adolescence, especially between the ages of 10 and 14–15 [2,11]. In 2021, the global incidence of T1DM among children and adolescents under 15 was 651,700 cases, with 108,200 new cases reported annually. This upward trend is attributable to elevated incidence rates and reduced mortality [19].

The underlying pathophysiology of T1DM is the destruction of pancreatic β-cells, resulting in a deficiency in insulin production [11,15,20]. The management of this condition necessitates rigorous blood glucose monitoring, insulin therapy, diabetes education, medications, regular physical activity, and dietary control [21,22]. Without adequate management, T1DM can lead to complications affecting various organs and systems, increasing the risk of severe and potentially life-threatening conditions [2,23]. Furthermore, the impact on the quality of life of adolescents with T1DM is significant [17].

The challenges associated with Type 1 Diabetes Mellitus (T1DM) extend beyond the physical health domain, as the condition has also been linked to a range of psychosocial difficulties experienced during adolescence [20]. Research has demonstrated that robust social and family support networks, in conjunction with a positive self-concept, can function as protective factors for adolescents with T1DM [24]. Furthermore, research indicates that more than 25% of children and adolescents diagnosed with chronic illnesses such as diabetes also have a diagnosed mental health condition [25], which increases their vulnerability to psychological disorders [26]. Emotional difficulties such as depression [27,28] and anxiety [6,7,29–31] are more prevalent among young people with diabetes. The presence of a chronic illness during adolescence has been associated with emotional and behavioural problems [9], as well as a decline in quality of life [20], both of which can interfere with treatment adherence and raise the risk of complications.

Adolescents diagnosed with chronic conditions such as T1DM have been observed to respond with denial or resistance to treatment [32]. Furthermore, as previously mentioned, adolescence is a period of increased risk-taking behaviours, which can lead to non-adherence to treatment plans and further exacerbate the likelihood of complications [6,17]. Given that there is currently no cure for T1DM, treatment is primarily aimed at improving quality of life, minimising risk factors, and reducing the number of complications [7,10,24].

The treatment of adolescents with diabetes is not solely focused on the delivery of medical care; rather, it is a multifaceted approach encompassing a range of indicators, including health-related quality of life [33–36] and physical and socio-emotional well-being [37–39]. Empirical evidence has demonstrated the efficacy of psychological interventions that cultivate emotional intelligence and assertive communication in enhancing well-being and quality of life in individuals with T1DM [40,41]. Such treatment programmes typically encompass domains such as adapting to the diagnosis, fostering emotional awareness and regulation, cultivating problem-solving skills, mitigating negative thought patterns and worry, nurturing positive beliefs, reducing psychological symptoms (particularly anxiety, depression and behavioural issues), and fortifying identity, self-concept and social skills.

In this context, information and communication technologies (ICTs) have emerged as effective tools for addressing various psychological and emotional needs [42]. Among these, serious games—defined as games or activities designed for purposes beyond entertainment, such as teaching skills or concepts [43,44]—have gained popularity. These tools are widely utilised in educational and therapeutic settings due to their interactive nature, which fosters high motivation and engagement [45,46]. In the realm of psychological interventions, these games have been incorporated to enhance patient understanding of chronic illnesses and promote emotional adjustment [43,47].

In the context of this study, serious games developed for children and adolescents with T1DM have proven effective in facilitating meaningful learning. These games have been shown to convey crucial knowledge about the condition, support skill development, and enhance motivation and self-efficacy, all of which have a positive impact on adjustment to living with T1DM [48].

### Objectives

The main objectives of this study are: (1) to design and implement a *serious game* called *emoTICare* to promote physical and emotional health, as well as the development of socio-emotional skills among adolescents with Type 1 Diabetes Mellitus (T1DM) through a psychological intervention conducted on a technological platform that incorporates artificial intelligence and Ecological Momentary Assessment (EMA); (2) to analyze the effectiveness of this *serious game* on indicators of disease adjustment, quality of life, and socio-emotional well-being, as well as improvements in socioemotional competencies and skills.

The study hypothesizes that adolescents will maintain or improve their physical and emotional health indicators following the *emoTICare* program. Specifically, statistically significant improvements (*p* ≤ .05) are expected in the following variables: health-related quality of life, social skills and communication, emotional awareness, resilience, self-esteem, as well as coping and problem-solving abilities. Additionally, a statistically significant reduction (*p* ≤ .05) is anticipated in variables related to the perceived threat of the disease, as well as emotional and behavioral problems. The experimental group is expected to show improvements in all these areas when comparing pre- and post-treatment evaluations, and also in comparison to the control group on the waiting list.

## Materials and methods

### Study design

A clinical longitudinal experimental design with inter-subject comparisons was employed, with the aim of comparing an experimental group (i.e., individuals receiving psychological treatment) and a control group on the waiting list (i.e., those without treatment) at three time points: before treatment (T1), and two post-treatment time points (T2, T3). The control group hasn’t any alternative activities or exposure to placebo-like interactions. Furthermore, an intra-subject analysis was conducted by comparing repeated measures to observe changes within the same individuals before (with pre-treatment measurements, T1), after 6 weeks (T2, in the control group and post-treatment in the experimental group), 6 months after T2 (T3, in the control group and post-treatment in the experimental group).

Initially, immediately after completing the informed consent online, a first evaluation will be conducted for all participants (T1), covering the main areas that constitute the psycho-emotional state of the participants. Following this first evaluation, participants in the experimental group will engage in six weeks of gameplay, with each week dedicated to completing one mission of *emoTICare*. At the six-week mark after the initial evaluation, and immediately following the completion of the *emoTICare* application by participants in the experimental group, a second evaluation (T2) of all participating adolescents (control and experimental groups) will take place. Following a period of six months from T2, a subsequent measurement, designated T3, will be conducted in all participants.

T1 will serve as the baseline measure of the psycho-emotional state of participating adolescents before the commencement of the *emoTICare* intervention. The homogeneity of the control group will be evaluated by comparing the T1 scores of both the control and experimental groups to ensure no significant differences exist, thereby allowing for comparable study groups. T2 will also function as a control measure for all participants concerning the evaluated variables. However, in the control group, only the changes resulting from the natural progression of time without intervention will be examined, while in the experimental group, the benefits achieved in these variables following the *emoTICare* intervention will be analyzed. T3, the follow-up measure, will also function as a control measure after six months for all participants with regard to the evaluated variables.

This experimental study includes a randomized clinical trial aimed at assessing the effects of completing the *emoTICare* program concerning the socio-emotional competencies and psychological well-being of these adolescents. The method employed for generating allocation sequences was a computerized random number generator. The sequence generation was based on a process of simple randomization, founded upon a single sequence of random allocations. The R programme was employed to allocate the participants at random to distinct groups (control and experimental) with equal probability. Consequently, it was guaranteed that every participant had an equal probability of being placed in any group. In order to guarantee allocation concealment and minimise selection bias, the allocation process was conducted in a controlled and secure environment. The sequence generated by the R program was stored in such a way that the researchers responsible for allocation did not have access to allocation information prior to the inclusion of participants in the study. Furthermore, the allocation was executed by an independent researcher external to the team, who interacted directly with the participants, thereby facilitating the maintenance of blinding. The integrity of the procedure was ensured through regular monitoring, and any possibility of manipulation of the allocations was avoided. This ensured that both the participants and the researchers responsible for the intervention were blinded to the assigned group, thus minimising any possible selection bias and ensuring the validity of the results.

The principal investigator will be responsible for obtaining informed consent from the participants (adolescents and parents/legal guardians) by informing them about the study. Adolescents aged between 12 and 16 inclusive will be required to sign the consent form in conjunction with their guardian, while those aged 16 and over will be permitted to sign it directly.

The collection of data will be facilitated through the utilisation of the Limesurvey platform, wherein the participants will be prompted to complete questionnaires in an anonymous manner. The principal investigator will supervise access and data security, and the research team will only access anonymised data for analysis.

The research team will measure baseline data at the commencement of the study through online questionnaires, and also the two post-test measures. The randomisation process will be overseen by the principal investigator and team, and will be conducted using a computerised random number generator.

The research team will be responsible for configuring and training participants and staff on the use of the Limesurvey platform, ensuring proper usage of the system.

**Fig 1:**
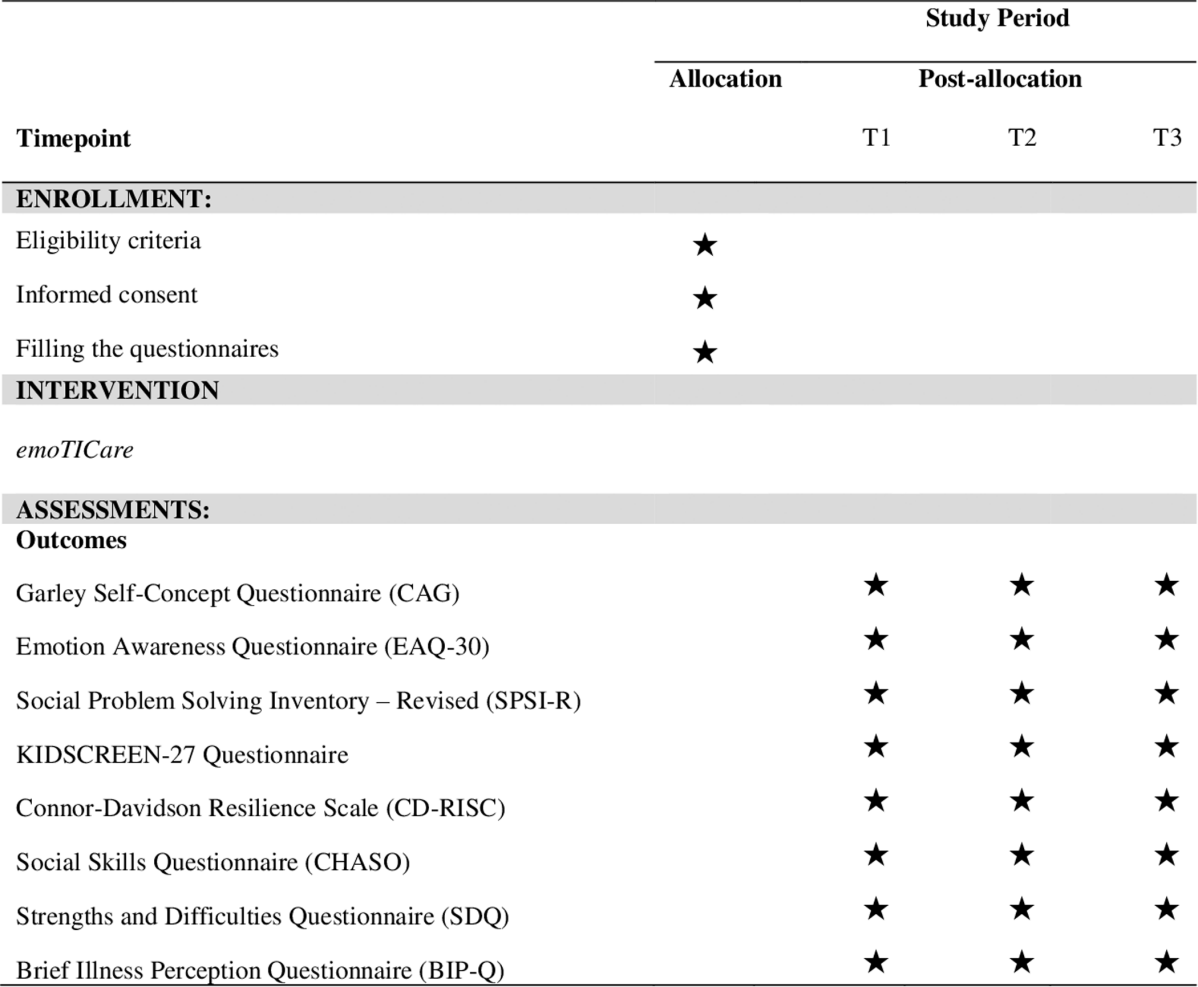
SPIRIT schedule of enrolment, interventions and assesments.

**Fig 2:**
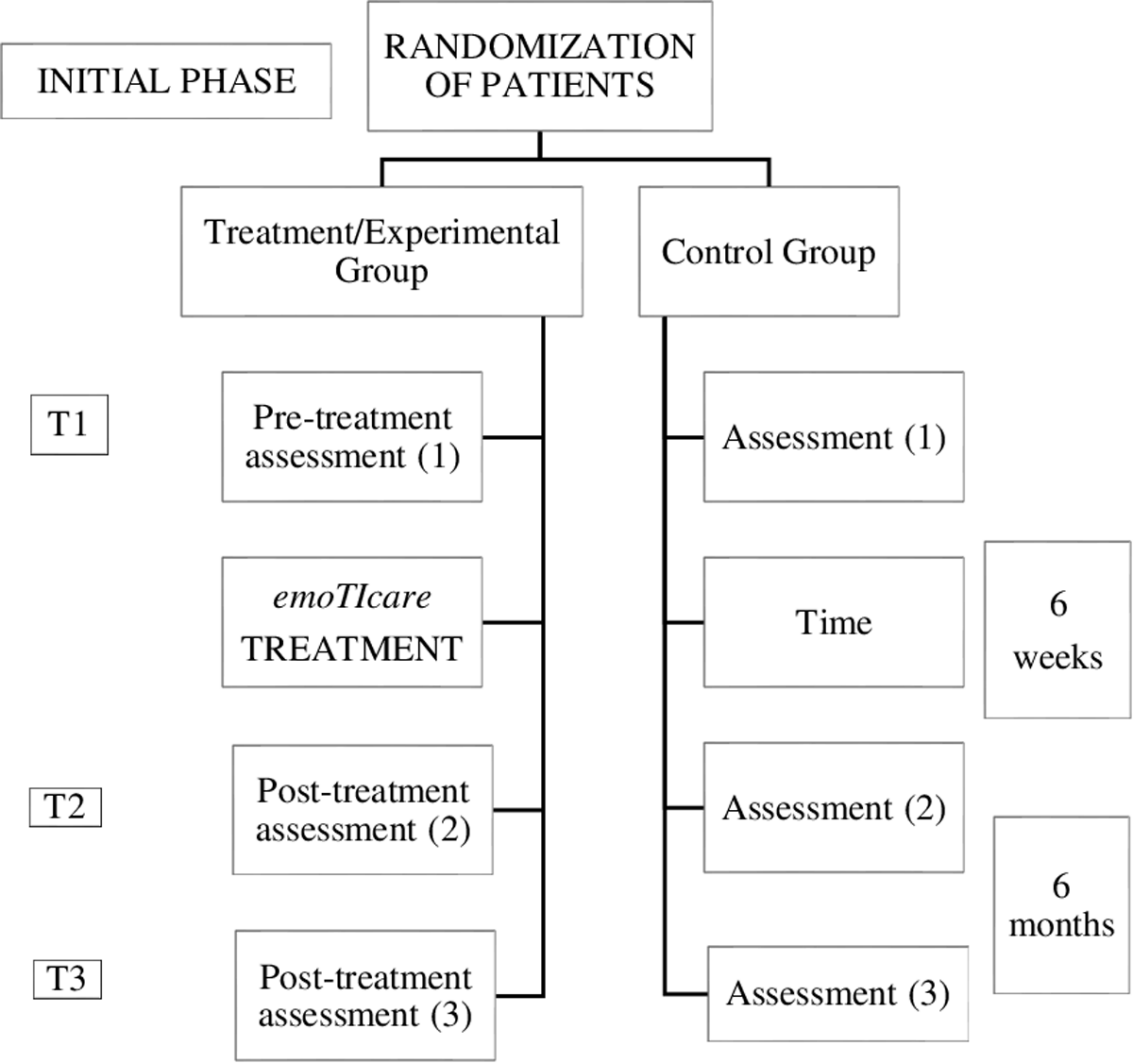
Study design of the serious game intervention.

According to the Standard Protocol: Recommendations for Interventional Trials statement [49], the current study protocol outlines the details of the study rationale, objectives, interventions, methods and statistical analyses, organization, and ethical considerations (See Figs 1 and 2).

The literature review process, the design of the psychological content of *emoTICare*, and the technological design of the *serious game* were conducted from December 2022 to June 2024. In July and August 2024, a beta study (pilot study) was carried out with a smaller sample of adolescents without Type 1 Diabetes Mellitus, during which the *emoTICare* program was tested to correct any potential errors or issues that arose during gameplay, as well as to gather feedback from the adolescents. Since September 2024, recruitment has commenced for the study sample of adolescents with Type 1 Diabetes Mellitus.

An accidental or convenience sampling method was employed. Participants were recruited as follows: all diabetes-related associations in Spain (over 100) were contacted via email and/or phone to request their voluntary participation in the study. These associations were asked to disseminate the study information to their members so that interested individuals could contact the researchers via email to participate. Regarding potential confounding variables, we acknowledge that differences between groups may arise due to the non-randomized recruitment method. To address this, we collected demographic and clinical variables (e.g., age, gender, diabetes type, disease duration) to assess and statistically control for potential confounders in the analyses.

In terms of selection bias, the recruitment process relied on voluntary participation through patient associations, which may lead to an overrepresentation of individuals who are more engaged in diabetes-related activities and support networks. This potential bias was mitigated by reaching out to a broad range of associations across Spain, ensuring geographic and organizational diversity in participant recruitment.

The phase encompassing the evaluation of the variables of interest, T1-T2-T3, will take place through the survey platform of the Universitat de València, Limesurvey, from September 2024 to the end of February of 2027. The response platform for the questionnaire battery includes a consent form for anonymous participation in the study. In this platform, informed consent will be obtained from the parents or legal guardians of the participants, as well as from the minors themselves, ensuring that all are informed about the anonymity and confidentiality of their data, confirming that their participation in the study is voluntary.

To access and play the *emoTICare serious game* online for six weeks (one week per mission of the game), participants will be provided with a link at the end of the evaluation survey conducted at T1. This *serious game* contains six distinct areas, each designed to be completed within a maximum of one week, thus allowing sufficient time to progress at different paces. During the period in which the adolescents are completing the *serious game*, an email address and a mobile phone number will be made available for participants to contact in case they need assistance (see Table 1).

**Table 1:** Intervention timeline.

				Timeline (in months)																															
	2024							2025												2026												2027			
	Jun	Jul	Aug	Sep	Oct	Nov	Dec	Jan	Feb	Mar	Apr	May	Jun	Jul	Aug	Sep	Oct	Nov	Dec	Jan	Feb	Mar	Apr	May	Jun	Jul	Aug	Sep	Oct	Nov	Dec	Jan	Feb	Mar	Apr
**Phase I**																																			
Enrollment and sample	★	★	★	★	★	★	★	★	★	★	★	★	★	★	★	★	★	★	★	★	★	★	★												
Assessment T1				★	★	★	★	★	★	★	★	★	★	★	★	★	★	★	★	★	★	★	★	★	★	★									
**Phase II**																																			
Control Group																																			
Experimental Group (*emoTICare* implementation)						★	★	★	★	★	★	★	★	★	★	★	★	★	★	★	★	★	★	★	★	★	★	★							
**Phase III**																																			
Assessment T2								★	★	★	★	★	★	★	★	★	★	★	★	★	★	★	★	★	★	★	★	★							
Preliminary data analysis																				★	★	★	★	★	★	★	★								
Dissemination of preliminary results																					★	★	★	★	★										
**Phase IV**																																			
Assessment T3													★	★	★	★	★	★	★	★	★	★	★	★	★	★	★	★	★	★	★	★	★		
Final data analysis and dissemination																												★	★	★	★	★	★	★	★

### Sample size

In Spain, the report on “Prevalence of Diabetes Mellitus” [50] highlights that more than three million people in the country have diabetes, of which 3.4% have Type 1 Diabetes Mellitus (T1DM). The Spanish Diabetes Society [51] estimates that T1DM accounts for 1 in every 10 cases in Spain, totaling approximately 90,000 individuals with this condition. Specifically, the incidence of diabetes in individuals under 15 years old varies between 11 and 24 cases per 100,000 inhabitants per year [51].

To determine the sample size, we used a 95% confidence level and a 5% margin of error, which is a standard approach in medical and epidemiological research. This calculation was based on the estimated population of individuals with T1DM under 15 years of age (approximately 90,000 individuals). Considering the anticipated dropout rate and the expected effect size, the total sample size was calculated to be 372 participants. This estimate assumes a normal distribution of the population and that the outcomes of interest are representative of the general T1DM population in Spain. Assumptions made during the calculation include: A 95% confidence level and 5% margin of error; A normal distribution of the studied variables, consistent with similar research in the field.; A moderate effect size based on previous studies investigating similar interventions and a 20% dropout rate, which is typical for this type of intervention study.

Limitations of the sample size calculation include potential variability in the effect size, which could differ from our assumptions. Additionally, since participants are recruited through specific diabetes associations, this sample may not fully represent the broader T1DM population, potentially introducing selection bias. Given the potential variability in healthcare access, lifestyle factors, and diabetes management across different regions, participants’ region of origin was recorded to assess potential regional differences. This information allows us to explore whether geographical factors may influence the study outcomes and, if necessary, include region as a covariate in statistical analyses to control for potential biases due to regional heterogeneity.

### Eligibility and recruitment criteria

To be included in this study, participants must be aged between 12 and 16 years, have a diagnosis of Type 1 Diabetes Mellitus (T1DM) for at least six months, not have a diagnosis of any other chronic physical illness, and not have any prior diagnosis of psychological disorder. Additionally, they must have completed the informed consent form prior to participation.

Exclusion Criteria: Physical diagnosis other than type 1 diabetes mellitus; No access to internet or new technologies; Not speaking Spanish.

The opportunity to participate in the study, along with relevant information will be communicated through various channels. In order to recruit participants, the study will be publicized throughout Spain through the dissemination channels of diabetes associations. To participate, parents or legal guardians of the adolescents must provide prior written informed consent.

Regarding the status and timeline of the study (we offer an estimate): A) participant recruitment will be completed by April of 2026; B) data collection will be completed by February of 2027 and C) final results are expected by April of 2027. None of these stages have already been completed.

### Interventions

The *emoTICare* technological platform intervenes with participants through a *serious game*, which is defined as a video game primarily designed to teach or provide the player with certain skills that serve to achieve a purpose beyond the game itself [43,44]. Accordingly, using the theoretical model of chronic illness adjustment developed, a series of playable areas were created that address the main factors promoting adaptive adjustment to chronic illness, specifically Type 1 Diabetes Mellitus in the adolescent population.

Firstly, when selecting the narrative context of the game, the aim was to generate a certain degree of immersion, as this has been shown to increase the likelihood of changing beliefs and attitudes in the real world [52].

### Development of the platform

Health-related quality of life (HrQoL) is a multidimensional construct that encompasses the physical, emotional, and social aspects of well-being. Unlike general quality of life, HrQoL primarily refers to the effect that illness and its subsequent treatment have on the various functional areas of an individual [53–55]. As previously explained, adolescence is a stage of life marked by critical changes that require personal skills to manage. Given the importance of coping with a chronic illness such as Type 1 Diabetes Mellitus (T1DM) during this period, and the need to promote socioemotional resources during this developmental transition to enhance adolescents’ quality of life, an initial review of the existing scientific literature on conceptual models of illness adjustment and health prevention was conducted. This review sought to identify common indicators of optimal positive development during adolescence, establishing a framework to promote adolescent quality of life.

As a result, a model was developed—the *Disease Adjustment Model from an Integrative Perspective* (DAMIP) [56]—which integrates a solid theoretical framework to facilitate the design, justification, and development of psychological interventions aimed at preventing psychological difficulties and enhancing adjustment to chronic illness during adolescence, thus improving quality of life and emotional well-being in this population.

The theoretical model was developed through the integration of different concepts and indicators present in pre-existing theoretical models, leveraging their complementarity. DAMIP draws upon Livneh’s Integrative Model [57,58], supplemented by Antonovsky’s Salutogenic Model [59], Leventhal’s Self-Regulation Model of Illness [60], and Hochbaum’s Health Belief Model [61], as well as elements from the proposal for promoting quality of life in adolescents from the National Academies of Sciences, Engineering, and Medicine (Health and Medicine Division; Division of Behavioral and Social Sciences and Education; Board on Children, Youth, and Families; Committee on Applying Lessons of Optimal Adolescent Health to Improve Behavioral Outcomes for Youth), led by Kahn and Graham [62], and the Kidscreen Project by Ravens-Sieberer et al. [63,64]. The DAMIP model provides the scientific community with a theoretical framework for optimizing socioemotional diagnosis and intervention in this population.

Based on the theoretical configuration of the DAMIP model, its core elements were incorporated into the development of the *emoTICare serious game*. The game consists of six missions aimed at fostering the most frequently cited concepts related to the structure underlying optimal development and well-being, which are crucial when implementing interventions to improve the biopsychosocial health of adolescents.

### emoTICare

The *emoTICare* program engages users through a serious game aimed at developing socio-emotional skills and promoting physical and emotional health in adolescents with T1DM. This graphic adventure game consists of a time-travel journey across different parts of the world and various historical periods. Using the DAMIP model, a series of playable areas were developed to address the key factors that promote adaptive adjustment to chronic illness, specifically tailored to T1DM in the adolescent population.

According to the principles of *serious games*, players need to experience a certain level of immersion, as this increases the likelihood of changing beliefs and attitudes in real life [52]. To achieve this, a narrative context was established to enhance immersion, supported by a fictional storyline and well-defined goals that guide the player through the challenges necessary to complete the intervention.

After reviewing possible genres and settings and consulting the target population about their preferences, it was concluded that the storyline and setting should feature a graphic adventure incorporating time travel mechanics.

Initially, the player awakens disoriented in an unfamiliar place, which turns out to be the Temporal Control Center. Here, they meet an Artificial Intelligence called *Alura*, who oversees the center and will serve as their guide to help them understand the game mechanics.

*Alura* explains that a temporal rupture has occurred, causing the clock of time to stop, and its cores have been scattered in unknown places, altering the world as it was known. From this point on, the character embarks on a space-time adventure to recover all the cores and repair the clock (for more detailed information on emoTICare, access the S1 File).

Throughout the game, the player will travel to six distinct areas: Egypt (Psychoeducation Area), Venice (Emotional Awareness Area), the Amazon (Emotional Regulation Area), *NewGlobal* (Cognitive Coping Area), Petra (Identity Area), and India (Social and Communication Skills Area).

Each of these areas will focus on one of the indicators of quality of life in adolescents specified in our theoretical model, as follows:

#### Area 1: Psychoeducation about the disease.

This area focuses on the physical well-being of the user and is designed to provide psychoeducation on T1DM, ensuring users gain a deeper understanding of their illness, which in turn promotes better adjustment. This area comprises four main tasks: Use of the Glucometer, Dietary Control, Physical Exercise, and Basic Knowledge of Type 1 Diabetes.

#### Area 2: Emotional awareness.

In this area, the foundation for the user’s emotional well-being is established. First, users must become aware of both their own emotions and those of others. Therefore, this area covers the primary definitions and functions of basic emotions (joy, fear, etc.) and complex emotions (love, shame, etc.), as well as the recognition and identification of these emotions in themselves and others. It consists of five tasks: Utility and Definition of Emotions, Identification of Emotions, Definition of Complex Emotions, Emotional Vocabulary, and Emotions in Context.

#### Area 3: Emotional regulation.

This area continues to focus on the user’s emotional well-being while introducing strategies for emotional coping. It emphasizes the regulation and control of various emotions, along with relaxation techniques. This area consists of two main tasks (Cognitive Relaxation/Mindfulness and Muscle Relaxation) and one ongoing task (Knowledge and Practice of Short-Term Stress Management Techniques). The user will learn seven short-term stress control strategies and determine which to employ in response to various situations encountered throughout the area. The strategies include: Abdominal Breathing, Internal Distraction, External Distraction, Internal Dialogue, Cognitive Discharge, Motor Discharge, and Environmental Change. Ultimately, the aim is to provide the user with effective tools for managing stress in real-life situations.

#### Area 4: Cognitive coping.

After addressing emotional well-being and coping strategies, users advance to learning tools for cognitive coping in potentially stressful situations. This area comprises three essential tasks: Problem-Solving, Self-Instruction Training, and Thought Stopping and Cognitive Restructuring.

#### Area 5: Identity.

This area focuses on the user’s self-perception and the values that define them, aiming to highlight concepts such as self-esteem, self-concept, self-image, and self-awareness. The tasks in this area are: Personal Qualities, Values, Self-Reflection Questions, and Shadow Test.

#### Area 6: Social development and communication.

Finally, the last area centers on the user’s social skills and how they express and present themselves to others. Concepts such as assertiveness, conflict coping styles, and barriers to effective communication between two individuals will be explored. The four tasks in this area are: Positive Communication, Active Listening, Assertiveness and Social Skills, and Conflict Resolution.

### Minigames

In addition to the areas that users will progressively access throughout the intervention, specific tasks have been developed for each area. These tasks aim to ensure users continue practicing the acquired techniques and reinforce their learning. The first five areas will feature a repeatable “minigame” that users can play as many times as they like while waiting for the next area to become available. The minigames are: Knowledge of Diabetes, Emotion Identification, Relaxation Techniques, Key Point Analysis, and Changing Irrational Beliefs about Identity.

### Assessment tools

#### Clinical and sociodemographic variables.

Sociodemographic variables such as gender, age, nationality, previous contact with psychology professionals, and reason for consultation will be assessed using an ad-hoc record after informed consent has been obtained.

Additionally, to gather relevant data related to the illness and its impact on adolescents, an ad-hoc record will collect the following medical variables:

- Glycated Hemoglobin (HbA1c)
- Cause and number of hospitalizations due to diabetes
- Months since the onset of the disease
- Perceived difficulty due to the illness
- Specific aspects of diabetes causing the most difficulty
- Presence of other medical conditions
- Presence of psychological disorders
- Type of medical treatment received for T1DM
- Physical consequences of T1DM

#### Psychological variables.

To assess the **perceived threat of disease**, the ***Brief Illness Perception Questionnaire*** (BIP-Q) by Broadbent et al. [65], adapted for Spanish adolescents ([66], will be used. This questionnaire includes five Likert-type items (0–10), along with an open-response item. It evaluates cognition through subscales for *Consequences of the Disease*, *Duration of the Disease*, and *Identity*, as well as emotional aspects through subscales for *Concern about the Disease* and E*motional Impact of the Disease*. The open-ended item assesses the causal representation of the disease. The adapted version shows satisfactory psychometric properties, with reliability of α = 0.76 and good internal validity.

**Health-related quality of life** will be measured using the validated Spanish version of the ***KIDSCREEN-27*** questionnaire [67]. This instrument consists of 27 Likert-type items (1–5), divided into five dimensions: *Physical Well-Being*, *Psychological Well-Being*, *Autonomy and Parent Relations*, *Social Support and Peers*, and *School Environment*. The original version of the questionnaire has good internal validity and high Cronbach’s alpha scores above 0.70 in the five dimensions. The Spanish version also demonstrates acceptable reliability and construct validity, with internal consistency scores equal to or greater than 0.70 across all dimensions.

**Psychopathology** will be assessed using the ***Strengths and Difficulties Questionnaire*** (SDQ) by Goodman [68], validated in Spanish by Ortuño-Sierra et al. [69]. This tool evaluates emotional and behavioral symptoms, as well as prosocial behavior in children and adolescents. It consists of 25 Likert-type items (0–3), grouped into five scales: *Emotional Symptoms*, *Behavioral Problems*, *Hyperactivity*, *Peer Problems*, and *Prosocial Behavior*. The first four scales assess behavioral and emotional difficulties, while the fifth focuses on positive social behaviors. The sum of the first four scales (excluding *Prosocial Behavior*) forms a sixth scale, *Total Difficulties*. The self-report version of the questionnaire has demonstrated adequate internal consistency in most studies. In its adapted Spanish version [69], internal consistency scores for the various subscales range from 0.58 (*Behavioral Problems*) to 0.71 (*Emotional Symptoms*).

To assess **social skills** and **communication abilities** in adolescents, the latest validated version of the *Social Skills Questionnaire* (CHASO) by Caballo et al. [70] will be used. It comprises 40 Likert-type items (1–5) and evaluates 10 skills: *Interacting with Strangers*, *Expressing Positive Feelings*, *Facing Criticism*, *Interacting with People Who Attract Me*, *Staying Calm in the Face of Criticism*, *Public Speaking/Interacting with Superiors*, *Dealing with Embarrassment*, *Defending One’s Rights*, *Apologizing*, and *Rejecting Requests*. Psychometric properties show a reliability of α = 0.88 and a Guttman reliability coefficient of 0.86.

**Emotional awareness** will be evaluated using the ***Emotion Awareness Questionnaire*** (EAQ-30) by Rieffe et al. [71], adapted into Spanish by Samper-García et al. [72]. This 30-item Likert-type questionnaire (1–3) identifies how adolescents perceive their emotions, the intensity with which they feel them, and their ability to express them. It comprises six subscales: *Emotion Differentiation*, *Verbal Sharing*, *Not Hiding Emotions*, *Body Awareness*, *Emotion Analysis*, and *Attention to Others’ Emotions*. The validation of the scale in a Spanish sample yielded a reliability score of α = 0.74.

To evaluate **coping and problem-solving skills** in adolescents with T1DM, the short version of the ***Social Problem Solving Inventory – Revised*** (SPSI-R) by Zurilla and Nezu [73] will be used. This questionnaire measures cognitive, emotional, and behavioral responses to everyday problems or difficulties. It consists of 25 Likert-type items, grouped into five dimensions: *Rational Problem Solving* (RPS), *Avoidant Style* (AS), *Impulsive Style* (IS), *Positive Problem Orientation* (PPO), and *Negative Problem Orientation* (NPO). The questionnaire has demonstrated adequate psychometric properties [74], with reliability scores for the five dimensions as follows: RPS (α = 0.76), AS (α = 0.74), IS (α = 0.74), PPO (α = 0.67), and NPO (α = 0.76).

**Resilience** will be measured using the 10-item ***Connor-Davidson Resilience Scale*** (CD-RISC) [75], validated in Spanish by Notario-Pacheco et al. [76]. This scale uses Likert-type items (0–4) and has demonstrated strong psychometric properties (α = 0.85).

Adolescent **self-concept** will be assessed using the latest version of the ***Garley Self-Concept Questionnaire*** (CAG) by García Torres [77]. This instrument aims to measure self-concept from a multidimensional perspective in adolescents. It consists of 48 Likert-type items, providing a global self-concept score, and is divided into six subdimensions: *Physical Self-Concept*, *Social Acceptance, Family Self-Concept*, *Intellectual Self-Concept*, *Self-Evaluation*, and *Sense of Control*. The questionnaire shows adequate psychometric properties for both the global score (α = 0.87) and the subscales.

Lastly, as a control variable, the ***Oviedo Infrequency Response Scale*** will be administered, developed by Fonseca-Pedrero et al. [78]. This scale consists of 12 Likert-type items (1–5) that are distributed throughout the questionnaire at key points. The items are organized into three groups of four, coded according to the questionnaire variable in which they are integrated. If a subject answers three or more items incorrectly, their questionnaire responses will be invalidated.

### Measurements

Missing data will not be an issue, as the survey is conducted through LimeSurvey, where all questions are mandatory and cannot be skipped by participants.

To be included in the data analysis, participants must have completed all three measurement points and answered all questionnaires.

T1: First measurement: After the participant had signed the informed consent, the initial score for this outcome was assessed.
T2: Second measurement: POST Up to 6 weeks after the second measurement, the second measurement of the same variable was carried out.
T3: Third measurement: POST Up to 6 months after the second measurement, the third measurement of the same variable was carried out.

[Time Frame: Baseline up to 30 weeks]

Primary Outcome Measure:

1. Change Quality of Life (measured by KIDSCREEN-27).
2. Change Emotional and Behavioral Problems (measured by SDQ).
3. Change Emotional Competences (measured by EAQ-30).
4. Change Self-concept (measured by CAG)
5. Change Coping (measured by SPSI-R).
6. Change Social Skills (measured by CHASO).

Secondary Outcome Measure:

1. Change Threat of disease (measured by BIP-Q)
2. Change Psychoeducation Diabetes

Psychoeducation of the disease: the ECODI Diabetes knowledge scale will be used to assess this variable [79]. This scale consists of 25 items and four dimensions: basic knowledge and self-care, laboratory values, diet and physical exercise. It is a scale that is corrected by the number of hits and misses.

[Time Frame: Start of the game and during the game]

1. Change Resilience (measured by CD-RISC).

Other Pre-specified Outcome Measures:

1. Glycaemic control

HbA1c: It will be used to evaluate the glycemic control of the diabetic patient in endocrinology diseases.

### Data analysis

In the present study, the per-protocol analysis (PP) approach was utilized to evaluate the effectiveness of the treatment and the management of missing data. This method includes only participants who fully adhered to the study protocol, ensuring that the results reflect the true efficacy of the intervention under ideal conditions. Participants will be included in the main analyses only if they have completed the treatment as per the protocol, that is to say, if they have adhered to all instructions without significant interruption. Participants who withdraw or fail to complete the treatment will be excluded from the analyses. This methodological decision is underpinned by the principle of minimizing potential bias from missing data, thereby ensuring that the results of the study accurately reflect the outcomes of participants who adhered to the treatment protocol. To address the issue of missing data during follow-up or treatment, predefined procedures will be implemented. The last observation carried forward (LOCF) imputation method will be employed to address missing data, with the last available measurement being utilised to complete the missing values. Furthermore, sensitivity analyses will be conducted to assess the impact of missing data on the study outcomes. These procedures are designed to minimise data loss, maintain the internal validity of the study, and ensure the robustness of the results.

Several statistical analyses will be performed to determine the effectiveness of *emoTICare*. As a first step, chi-square tests and t-tests were performed to examine potential differences between the control and experimental groups in key demographic and clinical variables (e.g., age, gender, disease duration). Although randomization is expected to ensure comparability, these tests were conducted to verify baseline equivalence. Propensity Score Matching (PSM) was not used to adjust for baseline differences, as the study followed a randomized controlled design.

Additionally, chi-square tests will compare the number of participants who withdraw from the experimental group with those who withdraw from the control group, ensuring no significant differences exist.

The primary focus of this study is on the evaluation of the programme’s overall effectiveness, as opposed to the presentation of definitive assertions concerning the outcomes of individual participants. It is imperative to emphasise that the tests are embedded in pre-specified models, with the primary focus being on assessing the overall effectiveness of the programme.

Before testing the change models, descriptive analyses, Pearson correlations, and multivariate and univariate analysis of variance (MANOVA and ANOVA) will be conducted using pre-intervention scores for participants in experimental and control groups to identify potential baseline differences. Furthermore, multivariate and univariate analysis of covariance (MANCOVA and ANCOVA) will be performed to detect changes in post-intervention scores (short-term effect), with pre-intervention scores as a covariate. These analyses will focus on latent variables derived from the composite scores of multiple Likert items, which represent underlying constructs such as emotional symptoms or health-related quality of life. Effect sizes (Cohen’s *d*) for each variable will also be calculated to estimate the magnitude of differences between the experimental and control groups. These analyses will be conducted using SPSS V.28.

To ensure the reliability of the results, we will begin with a series of diagnostic tests and validation procedures. This will include an analysis of residuals to assess the goodness of fit of the model, using residual plots and tests for normality (e.g., Q-Q plots and Shapiro-Wilk test). Homoscedasticity will be evaluated through the Breusch-Pagan or White’s test, and the Durbin-Watson statistic will be used to check for autocorrelation in the residuals. These tests will allow us to ensure that the assumptions of the parametric models are met. Additionally, we will conduct cross-validation (k-fold) to ensure that the model generalizes well to unseen data and is not overfitted. We will also perform outlier detection using Cook’s distance and leverage plots to examine the influence of individual data points on the model.

Multiple hierarchical regression analyses will be conducted to examine the added predictive value of the experimental condition in explaining the intervention program’s efficacy. Hierarchical regression is a stepwise method where predictors are added to the model in blocks. The rationale behind the hierarchical approach is to assess the incremental contribution of each new block of predictors while controlling for the variables entered in the previous blocks. This allows for a clearer understanding of the unique variance explained by the experimental condition, after accounting for initial factors.

Step 1: The first block typically includes variables measured at time 1 that are believed to influence the dependent variable (i.e., the change between T2-T1 and T3-T1 for each variable). These could include baseline measures of emotional symptoms, quality of life, or disease-related factors.

Step 2: In subsequent blocks, new independent variables (predictors) are added. For example, in the second block, we would add the experimental condition (treatment vs. control) to assess its impact on the change in the dependent variable, controlling for the baseline scores. The dependent variable will be the change between pre- and post-intervention scores, as well as between pre-intervention and follow-up scores. A statistically significant change in the coefficient of determination in Model 2 (predictor: experimental condition, controlling for pre-intervention score) will be interpreted as added predictive value. A significant prediction from the experimental condition, controlling for pre-intervention score, will allow attribution of significant change to the intervention.

The Reliable Change Index (RCI) will be calculated for the scores of the evaluated variables. Reliable change will be determined by the extent to which an individual’s progress exceeds the natural fluctuation of measurements [80]. The RCI will be calculated using pre- and post-*emoTICare* means, along with the pre-intervention standard deviation and Cronbach’s alpha index.

Following completion of the *emoTICare* program, statistically significant improvements (*p* ≤ .05) are expected in scores related to health-related quality of life, social and communication skills, emotional awareness, resilience, self-esteem, as well as coping and problem-solving skills. Conversely, a statistically significant reduction (*p* ≤ .05) is anticipated in variables associated with perceived disease threat, as well as emotional and behavioral problems.

An independent data safety monitoring board (DSMB) is to be convened at six-month intervals in order to review serious adverse event (SAE) reports. Should the DSMB determine that there is no association between the serious adverse events and the intervention under evaluation, the study may proceed. Conversely, if the events are found to be associated with the intervention, the DSMB is able to implement measures to ensure the safety of participants. In the event of the identification of serious adverse events, the DSMB may take the following actions: to halt the study, modify the protocol, adjust the treatment, enhance monitoring, or recommend early termination, with the overarching aim of ensuring participant safety.

### Ethics and dissemination

This study adheres to the ethical guidelines of the 2013 World Medical Association Declaration of Helsinki and has received approval from the Human Research Ethics Committee of the Universitat de València (Reference 2023-PSILOG-3178945, UV-INV_ETICA-3178945). Appropriate measures have been taken to ensure the complete confidentiality of participants’ data, under the Personal Data Protection Law (LOPD) 3/2018 of December 5.

This study and its protocol have been approved and registered as a clinical trial in the ClinicalTrials.gov PRS (Protocol Registration and Results System) (Reference – ID: EmoTICare NCT06331429).

This protocol follows the Standard Protocol Items: Recommendations for Interventional Trials (SPIRIT) 2013 guidelines.”

The results obtained will be disseminated to the main educational, social-health, and scientific stakeholders.

## Discussion

Type 1 Diabetes Mellitus (T1DM) is a chronic condition that significantly impacts the lives of adolescents diagnosed with it. The extensive treatment required, along with the consequences associated with the disease itself, can lead to both physical and psychological complications. Therefore, a multidisciplinary approach that not only includes medical treatment but also considers psychosocial aspects can improve the quality of life for these individuals. In this context, *serious games* present an alternative for implementing psychological intervention programs in a video game format, making it an engaging option for addressing important elements in a playful, accessible, and healthy manner while maintaining their educational purpose.

In this regard, the *emoTICare* program emerges as a psychoeducational intervention tool in electronic format, designed to indirectly address the daily challenges that many adolescents with T1DM face. This approach also aims to enhance treatment adherence and compliance.

This opens a pathway for practical and theoretical possibilities, allowing for the expansion of this work to other chronic diseases, which can be offered as a complementary approach to medical treatment and contribute to improving well-being and quality of life.

The results of this project represent a significant advancement in the development of emotional competencies and well-being in adolescents with T1DM. Regarding its scientific and technical impact, three key contributions of this research stand out: 1) it is a longitudinal study, with participants being evaluated at three stages; 2) it makes a significant contribution to the challenge of digital transition by incorporating new technologies into its framework and addressing social needs through problem-solving; and 3) it is a multidisciplinary (health and technology) and interdisciplinary (psychology and medicine) project. A dissemination plan is proposed for three target populations: a) the scientific community; b) participants; and c) social agents (educational, medical, and social institutions), at both national and international levels.

Implementing the program in hospitals and diabetes associations offers potential benefits for all stakeholders in the medical community and society at large, both psychologically and economically. This digital platform may positively affect the mental health of adolescents with chronic illnesses by fostering personal strengths that aid in adapting to their condition and the challenges of adolescence. Furthermore, there are economic benefits for the mental health system, as it may reduce the demand for short- and long-term care. For psychological health interventions to be effective, psychosocial factors associated with T1DM must also be promoted through appropriate protocols, such as the *emoTICare* intervention.

Despite the strengths of the study, several limitations exist. Firstly, a potential shortcoming of the present intervention is the restricted direct participation of family members, despite the pivotal function that familial assistance plays in the management of chronic illnesses during adolescence. While the programme is designed primarily with adolescents in mind, the serious game has been developed with the specific objective of promoting communication, active listening, social skills and conflict resolution. The overarching objective of these components is to nurture socio-emotional development and enhance interpersonal competencies, which are pivotal for fostering supportive relationships. Given the pivotal role of the family as a support system during this period of development, enhancing these skills is expected to engender more positive family dynamics and greater adherence to treatment. Nevertheless, future iterations of emoTICare could strengthen this aspect by incorporating structured communicative sequences within the game that explicitly address common challenges in family interaction. Such an expansion would facilitate a more integrated approach, enhancing the ecological validity of the intervention and supporting adolescents within their broader social context.

Secondly, incidental sampling may hinder the generalization of the program’s results, as the sample may not fully represent the wider population of adolescents with Type 1 Diabetes. Thirdly, the online and remote format of the evaluation process, along with the use of the emoTICare program, may present challenges in ensuring proper completion, potentially increasing the likelihood of experimental mortality. Moreover, the online format could impact participant engagement, as the lack of direct interaction may lead to decreased attention or motivation. This is particularly relevant as maintaining participant motivation throughout the study period is a challenge in long-term interventions, and participants may experience fatigue or loss of interest over time. Additionally, biases in self-reported data could arise, as participants might provide socially desirable answers or misinterpret the questions, affecting the accuracy of the data. Conversely, per-protocol analysis (PP) provides a clear understanding of the potential benefits of treatment by focusing on subjects who have completed their assigned treatment regimen. However, it is limited in that it does not account for real-world challenges to adherence in real-world settings.

A further limitation of this study is the absence of specific data on the incidence of type 1 diabetes in individuals under 16 years of age. Instead, the incidence in individuals under 15 was used as a reference. It is acknowledged that this may result in under- or overestimation, owing to the exclusion of data from 15–16-year-olds. It is also noted that, given the relatively narrow additional age span (one year), any deviation in incidence is unlikely to significantly affect the overall power or feasibility estimates of the study. Incidence figures for individuals under 15 years are extensively utilised in international diabetes surveillance and are regarded as robust and comparable across populations. Given that the majority of the target population falls within the 12–14 age range, it was considered reasonable to use incidence data for individuals under 15 years as a proxy for estimating the expected number of eligible participants. Furthermore, the incidence of type 1 diabetes between ages 15 and 16 does not markedly deviate from that observed in the immediately preceding years, particularly in populations with relatively stable incidence curves.

Finally, collecting a larger sample with more evaluations over time could provide better tracking of the changes achieved in adolescents after receiving emoTICare treatment, offering a more comprehensive understanding of its long-term effects.

## Supporting information

S1 Checklist SPIRIT Fillable checklist emoticare def. (DOC)

S1 File Supplementary information about emoticare. (DOCX)
