# Supplementary material for: Intervention through an intelligent technological platform for socio-emotional development and health promotion in adolescents with Type 1 Diabetes Mellitus (emoTICare): A study protocol for randomized controlled trial
Source: PLoS One. 2025 Jun 23;20(6):e0325763. doi: 10.1371/journal.pone.0325763 (PMC12184894; doi:10.1371/journal.pone.0325763)
Supplement: S1 File — (DOCX) [file pone.0325763.s002.docx]

# AREA 1. PSYCHOEDUCATION OF THE DISEASE

This area focuses on the physical well-being of the user, and in our case, it is the area in charge of providing psychoeducation on type 1 diabetes, so that users have a better understanding of their disease, which will provide a better adjustment to it. This area consists of 4 main tasks related to the principles of psychoeducation in diabetes:

- Use of the glucometer: The player must check their glucose level after each test, interacting directly with it on screen, thus developing the habit, and reinforcing the act of measuring their glucose level as something positive and necessary in their day-to-day life.
- Diet control: By means of a scale, the player will learn the glycaemic index of some of the most common foods and will be able to prepare a healthy and balanced dish from them, which will give the player an essential knowledge that will allow him/her to have a healthier diet.
- Physical exercise: This test highlights the importance of physical exercise for people with diabetes, as it is a great tool for regulating blood glucose levels. The player will have to pass 3 tests that mimic real physical wear and tear.
- Basic knowledge of type 1 diabetes: Finally, the player must know some of the basic terms related to type 1 diabetes, so in this test he/she will have to answer a series of questions with 3 alternatives to test his/her knowledge of the disease.

# AREA 2. EMOTIONAL AWARENESS

In this area we begin to lay the foundations for the emotional wellbeing of the user, and to do so, we must first make them aware of their own emotions as well as those of other people. Therefore, this area works on the main definitions and usefulness of basic emotions (joy, fear, etc.), as well as complex emotions (love, shame, etc.), and the recognition and identification of these emotions in oneself and in others. This area consists of 5 tasks:

- Usefulness and definition of emotions: The user should understand the definition and the physical and behavioural manifestations of the basic emotions, as well as the usefulness of the emotions.
- Identification of emotions: In this task the user should be able to identify basic emotions by observing only the facial features of a person, in this case, masks representing those features.
- Definition of complex emotions: Similar to the first task, the user should be able to identify the definition of some of the main complex emotions while observing their characteristics.
- Emotional vocabulary: The aim of this task is to expand users' emotional vocabulary. To do so, they will have to match the basic emotions we all know with their derivatives, which will have different degrees of intensity.
- Emotions in context: Finally, the user will have to recognise the expression of these emotions in different contextual situations that will be presented through the different characters of a puppet theatre.

# AREA 3. EMOTIONAL REGULATION

This area continues to work on the emotional well-being of the user and begins to provide emotional coping strategies. If the previous area focused on the facet of recognition and use of different emotions, this one focuses on their regulation and control, as well as relaxation techniques. This area is composed of 2 main tasks and 1 that is fragmented throughout the area:

- Short-term stress management techniques: The user will learn 7 short-term stress management strategies, and decide which of them to use in situations that will arise in the course. We hope that you will be able to put these techniques into practice when stressful situations occur in real life. These strategies are: abdominal breathing, internal distraction, external distraction, internal talking, cognitive unloading, motor unloading and environmental change.
- Cognitive relaxation/mindfulness: They will practice, through a diegetic audio, a narrated cognitive relaxation.
- Muscle relaxation: In the same way, they will do another muscle relaxation activity narrated through an audio.

# AREA 4. COGNITIVE COPING

Once they have worked on emotional well-being and coping, users will proceed to learn tools related to cognitive coping with potential stressful situations. This area is composed of 3 fundamental tasks:

- Problem solving: A problem will be posed to the user, who will have to follow the recommended steps to solve it, i.e. be able to define the problem and the people involved in the problem, generate possible alternative solutions and choose one of them using useful criteria, such as key point analysis. This will help the user to internalise the problem-solving process, so that he/she can apply it in real life.
- Self-instructional training: The user will then follow a replica of Meichenbaum's self-instructional training, thus strengthening his or her capacity for organisation, planning and attention to situations that may generate stress.
- Thought stopping and cognitive restructuring: Finally, the aim of this task is twofold; on the one hand, the user must check that there are negative intrusive thoughts that impact our ability to act and look for positive solutions, so he will be taught by examples that these thoughts are present and he must stop them. Once they have stopped them, they should be able to redirect their flow of thought and convert these irrational thoughts into more adaptive thoughts.

# AREA 5. IDENTITY

This area focuses on the user's perception of him/herself and the values that represent him/her, and aims to highlight concepts such as self-esteem, self-concept, self-image and self-knowledge. The tasks of which this area is composed are the following:

- Personal qualities: In this task the user is asked to choose, from a list provided by us, a number of qualities with which he/she feels comfortable, and with which to present him/herself to the world. In order to direct the expression of these qualities, the following questions are asked: "I like my...", "I am an expert in...", "With others I am...", "I consider myself to be..." and "I define myself as...". This task is intended to make you reflect on the skills and qualities you possess.
- Values: In this task the user will have to select a series of values to answer some questions. This task is similar in purpose to the previous one, the objective is to reflect on which values define the most important areas of your life, such as your family, or your friends. The questions to be answered are as follows. "What is the most important value in my life?", "What value represents me in my family?", "What value do I most identify with?", "What value do I most look for in others?", "What value represents me with my friendships?", "What value do I feel most strongly?
- Questions about yourself: You will be asked to answer the following questions about yourself, your concerns and some hypothetical situations. The questions are as follows: "For me life is...", "If I could change something I would be...", "If I were invisible...", "I would love to invent...", "I would love to do...", "If I could have a power I would be...".
- Shadow test: Finally, the user will have to arrange their thoughts, values, skills and likes and dislikes on their silhouette. The questions to be answered and placed on the different parts of the body are: "What do I think about myself?" "What emotions do I usually have?" "What are my main values?" "What are my main skills?" "What do I like or care about?" "What are my goals and desires?". In this way it is intended that the user sees a shadow of himself and can thus visually observe what he believes to be his identity.

# AREA 6. SOCIAL DEVELOPMENT AND COMMUNICATION

Finally, the last area will focus on the user's social skills, and how he/she expresses him/herself and exposes him/herself to others. In this way, concepts such as assertiveness, coping styles in the face of conflict and the barriers that prevent effective communication between two people will be worked on. The four tasks present in this area would be:

- Positive communication: The user will understand the concept of positive communication, through which we can express what we feel or want clearly, without affecting or harming others.
- Active listening: In the same way, in addition to knowing how to communicate properly, he/she must learn to listen actively, i.e. listening that is focused on the message of the interlocutor and that fosters empathy.
- Assertiveness and social skills: One of the key points in communication is social skills and the concept of assertiveness. In this task the user will learn the differences between passive, aggressive and assertive communication styles, and will be able to use the latter correctly.
- Conflict resolution: This last task will consist of differentiating and understanding the different conflict coping styles, such as: the avoidant style, the accommodative style, the competitive style, and the problem-solving style.

**Mini-games between areas.**

In addition to these areas, to which they will progressively have access throughout the intervention, tasks related to each of them have been developed, with the aim of allowing users to continue practising the techniques acquired and consolidate their learning. The first 5 areas will have a "mini-game" that can be repeated as many times as desired while the user waits for the next area to become available. These mini-games will be the following:

1. Knowledge of diabetes: The aim is for the user to review and continue learning facts related to type 1 diabetes mellitus. The user will be presented with a total of 25 questions with different alternatives, in groups of 5, and the user will have to select the correct alternative.
2. Identifying emotions: The user will be able to continue practising the recognition and definition of basic and complex emotions. They will have to recognise the characteristics of these emotions and relate them to each other.
3. Relaxation techniques: The objective is for the user to draw certain shapes on the screen of the device they are using following a pattern of patterned breathing, with the aim of encouraging relaxation. These shapes will change and become more complex as they are drawn several times.

4. +Key Point Analysis: In this mini-game, the user will practice the key point analysis technique, which will help him/her to discern whether the solutions he/she proposes to any of the problems he/she faces in real life are the most appropriate ones to solve that problem.

5. Motivational phrases about your identity: The task in this mini-game will be to transform phrases that reflect irrational beliefs about oneself, the world, and the future into ones that are motivational and promote or direct towards self- improvement.
